# Impact of estradiol-to-progesterone ratio before progesterone initiation on pregnancy outcomes in frozen embryo transfer cycles with hormone replacement therapy: an analysis of over 25,000 cycles

**DOI:** 10.3389/fendo.2026.1755013

**Published:** 2026-03-09

**Authors:** Jun Shuai, Weiwei Liu, Xiu Luo, Qi Zhang, Hong Ye, Guoning Huang

**Affiliations:** Center for Reproductive Medicine, Women and Children’s Hospital of Chongqing Medical University, Chongqing Health Center for Women and Children, Chongqing, China

**Keywords:** clinical pregnancy rate, estradiol-to-progesterone ratio, frozen-thawed embryo transfer, hormone replacement therapy, live birth rate

## Abstract

**Background:**

Endometrial receptivity in hormone replacement therapy–frozen embryo transfer (HRT-FET) cycles depends on a precisely coordinated estradiol–progesterone environment. An imbalance between estradiol (E2) and progesterone (P) before progesterone exposure may impair the endometrial transition from proliferation to secretory transformation. This study investigated whether the estradiol-to-progesterone (E2/P) ratio prior to the initiation of progesterone is associated with pregnancy outcomes.

**Methods:**

This retrospective cohort study analyzed HRT-FET cycles performed between 2017 and 2022 at a single reproductive medicine center. Serum E2 and P levels were measured prior to the initiation of progesterone, and the E2/P ratio was calculated to reflect their relative concentrations. Patients were categorized into quartiles according to their E2/P ratios. Multivariable and multi-model logistic regression analyses were conducted to evaluate the association between E2/P ratios and clinical pregnancy rate (CPR) as well as live birth rate (LBR).

**Results:**

Both CPR and LBR declined progressively with increasing E2/P ratios across quartiles (CPR: 60.6%, 57.7%, 52.7%, and 47.7%; LBR: 50.6%, 47.1%, 42.6%, and 36.7% from the lowest to highest quartile, respectively; P < 0.001). After adjusting for confounders including female age, body mass index, infertility diagnosis, infertility indication, embryo transfer stage, number of embryos transferred, and endometrial thickness, higher E2/P ratios remained independently associated with reduced CPR and LBR. The trend remained significant in all regression models (P for trend < 0.001).

**Conclusions:**

In HRT-FET cycles, higher pre-progesterone E2/P ratios were associated with lower clinical pregnancy and live birth rates. The E2/P ratio may reflect the hormonal status of the endometrium, but its clinical utility requires confirmation through prospective studies.

## Introduction

Successful embryo implantation requires precise synchronization between a developmentally competent embryo and a receptive endometrium (1). It is estimated that approximately one-third of implantation failures are attributable to embryonic factors, whereas the remaining two-thirds result from impaired endometrial receptivity or disrupted embryo–endometrium interactions (2). The ovarian steroids—estrogen and progesterone (P)—and their corresponding receptors (ER and PR) orchestrate a complex cascade of molecular and cellular events that establish uterine receptivity and support early pregnancy (3, 4). Under physiological conditions, estradiol (E2) promotes proliferation of endometrial epithelial and stromal cells and induces progesterone receptor expression, thereby priming the endometrium for subsequent progesterone-mediated secretory transformation. Progesterone, in turn, stimulates glandular secretion and stromal decidualization, processes essential for embryo implantation and early gestation (5). Disruption of this finely balanced endocrine milieu may lead to progesterone receptor downregulation or desensitization, impaired endometrial differentiation, and consequently, diminished implantation potential (6, 7).

With advances in cryopreservation and improvements in cumulative live birth rates, frozen embryo transfer (FET) has become an integral component of assisted reproductive technology (ART). Among FET regimens, hormone replacement therapy (HRT) cycles are widely employed because they permit flexible scheduling and precise control of endometrial preparation (8). However, HRT cycles rely entirely on exogenous steroid supplementation to mimic the natural endocrine milieu, which may not fully recapitulate the dynamic hormonal fluctuations observed in spontaneous cycles. Several studies have reported that excessively high or low serum E2 levels before progesterone initiation are associated with decreased implantation and live birth rates (9–12). Consistently, our previous large-scale retrospective study demonstrated that elevated serum E2 concentrations prior to progesterone administration in HRT-FET cycles were independently associated with lower clinical pregnancy and live birth rates (13). These findings highlight the importance of maintaining optimal hormonal balance to achieve favorable endometrial receptivity and implantation outcomes.

Recently, the estradiol-to-progesterone (E2/P) ratio has been proposed as an integrated indicator reflecting the relative balance between proliferative and secretory endometrial signaling. A small-scale study involving 35 patients reported that a higher E2/P ratio on the day before FET was associated with poorer pregnancy outcomes (14). In contrast, Zhao et al. observed that in fresh embryo transfer cycles, a higher E2/P ratio measured seven days after oocyte retrieval was associated with improved pregnancy outcomes, provided that the ratio remained within an appropriate range (15). Nevertheless, other studies—including randomized controlled trials and comparative analyses across fresh, HRT, and natural FET protocols (16–20)—failed to demonstrate significant associations between pre-progesterone hormonal status and reproductive outcomes. Such discrepancies likely reflect heterogeneity in study populations, sample sizes, and hormone administration protocols.

To date, clinical evidence on the relationship between the pre-progesterone E2/P ratio and pregnancy outcomes in HRT-FET cycles remains limited. Most existing studies have focused on absolute hormone levels or have been restricted to small observational cohorts, leaving the clinical implications of relative hormonal balance largely unexplored. Therefore, this study analyzed data from more than 25,000 HRT-FET cycles to systematically evaluate the association between the E2/P ratio prior to progesterone initiation—stratified by quartiles—and both clinical pregnancy rate (CPR) and live birth rate (LBR). Using multivariable logistic regression and trend analyses, we sought to determine whether the E2/P ratio serves as an independent predictor of reproductive outcomes. This large-scale analysis provides robust evidence to address a critical gap in the literature and may inform individualized hormonal monitoring strategies to optimize endometrial preparation and improve ART success rates.

## Methods

### Study design and population

This single-center retrospective cohort study analyzed data from the Center for Reproductive Medicine for all hormone replacement therapy–frozen embryo transfer (HRT-FET) cycles performed between January 2017 and December 2022. Embryo cryopreservation was exclusively performed using vitrification, and serum estradiol (E2) and progesterone (P) levels were assessed using a standardized hormone assay. To ensure consistency of hormone measurements, cycles with serum E2 levels >1,000 pg/mL prior to progesterone administration were excluded from the analysis (n ≈ 60), as these values were obtained after sample dilution using a different assay method that was not directly comparable to the primary measurements. Only cycles with E2 ≤1,000 pg/mL were included in the primary analysis. Patients were followed until live birth. Exclusion criteria included cycles involving patients with uterine malformations (unicornuate, septate, or didelphys uterus), untreated endometrial lesions (including endometritis, intrauterine fluid, endometrial polyps, intrauterine adhesions, or submucosal fibroids), uterine fibroids ≥5 cm in diameter, untreated hydrosalpinx, uncontrolled endocrine or immune disorders (e.g., thyroid disease, diabetes mellitus, systemic lupus erythematosus, or antiphospholipid syndrome), preimplantation genetic testing (PGT) cycles for chromosomal abnormalities, cycles involving gonadotropin-releasing hormone (GnRH) agonist treatment, embryos derived from *in vitro* maturation (IVM), or oocyte-donation cycles. Patients were categorized into four groups according to quartiles of the serum E2/P ratio prior to progesterone administration: Q1 <0.62, Q2 0.62–<1.14, Q3 1.14–<2.91, and Q4 ≥2.91. The E2/P ratio was calculated after converting progesterone units from ng/mL to pg/mL to match the units of estradiol.

### Endometrial preparation and luteal phase support

All patients underwent endometrial preparation with hormone replacement therapy. Treatment was initiated on cycle days 2–3 after confirming baseline serum E2 < 50 pg/mL and P < 1 ng/mL. Oral estradiol valerate (Progynova; Bayer-Schering Pharma AG, Berlin, Germany) was administered at 2–5 mg twice daily, with dosage adjustments based on endometrial thickness and morphology assessed by transvaginal ultrasonography on day 7 at the discretion of the treating physician. After 12–14 days of estrogen treatment, endometrial thickness and serum hormone levels were reassessed. If serum P remained <1 ng/mL, luteal phase support was initiated using vaginal micronized progesterone (Utrogestan, 200 mg three times daily; Besins Healthcare, UK). Embryo transfer was performed on day 4 for cleavage-stage embryos and day 6 for blastocysts, with the first day of progesterone administration designated as day 1 (D1). On the day of embryo transfer, oral dydrogesterone (Duphaston, 20 mg; Abbott, Netherlands) was added. Luteal support was continued until a positive β-hCG test and, if pregnancy was achieved, maintained until 12 weeks of gestation.

### Outcome measures

The primary outcomes were CPR and LBR, as defined by the American Society for Reproductive Medicine (ASRM, 2017) (21). Clinical pregnancy was confirmed by the presence of one or more gestational sacs on ultrasonography. Live birth was defined as the delivery of at least one viable infant beyond 22 weeks of gestation. CPR was calculated as the number of cycles with clinical pregnancy divided by the total number of embryo transfer cycles ×100. LBR was calculated as the number of cycles resulting in live birth divided by the total number of embryo transfer cycles ×100.

### Statistical analysis

Continuous variables are presented as mean ± standard deviation (SD), and categorical variables as frequencies and percentages. Differences between groups were compared using one-way analysis of variance (ANOVA) or Kruskal–Wallis tests for continuous variables, and chi-square tests for categorical variables. The predictive performance of the estradiol-to-progesterone (E2/P) ratio for clinical pregnancy was initially assessed by receiver operating characteristic (ROC) curve analysis, with an area under the curve (AUC) of 0.559, indicating limited discriminative ability; therefore, the ROC curve is not shown. As no clinically meaningful cutoff was identified, E2/P was analyzed both as a continuous variable and according to quartiles to capture potential nonlinear associations.

Multivariable logistic regression was used to identify independent predictors of clinical pregnancy rate (CPR) and live birth rate (LBR). Variables showing significant differences in univariate analysis were included. Collinearity was assessed using variance inflation factors (VIF) and correlation matrices, and endometrial thickness at the start of progesterone administration was retained in the final model due to collinearity with thickness at embryo transfer.

To evaluate the association of E2/P with pregnancy outcomes, three progressively adjusted multivariable logistic regression models were constructed: unadjusted, partially adjusted (for female age, body mass index, infertility type, and infertility indications), and fully adjusted (additionally adjusted for embryo transfer stage, number of embryos transferred, and endometrial thickness at the start of progesterone). To assess linear trends across quartiles, median values of each quartile were entered as continuous variables and a P for trend was calculated. Results are presented as adjusted odds ratios (aORs) with 95% confidence intervals (CIs). Two-sided P values <0.05 were considered statistically significant. Inverse probability of treatment weighting (IPTW) was additionally conducted as a sensitivity analysis to assess the robustness of the associations. For each pairwise comparison of higher E2/P quartiles (Q2–Q4) versus Q1, separate logistic regression models were used to estimate propensity scores based on all covariates in the fully adjusted model. Stabilized weights were derived from the predicted probabilities and applied using the “Weight Cases” function in SPSS. Weighted logistic regression was then performed to obtain IPTW-adjusted odds ratios and 95% CIs, which were compared with the multivariable model results for consistency. To further evaluate whether the associations were influenced by endometrial thickness distribution, a sensitivity analysis was conducted by restricting the cohort to cycles with endometrial thickness between 7–14 mm. Fully adjusted logistic regression models were rerun within this restricted sample, and aORs with 95% CIs were reported. Additionally, to examine the robustness of findings across different exposure classifications, the E2/P ratio was regrouped into tertiles, and fully adjusted logistic regression models were repeated using tertile categories. The resulting effect estimates were compared with quartile-based analyses to assess consistency. To directly compare the effects of serum E2 and the E2/P ratio within the same cohort, an additional augmented multivariable logistic regression model was constructed in which E2 quartiles and E2/P quartiles were simultaneously entered together with all covariates from the fully adjusted model. Collinearity between E2 and the E2/P ratio was assessed using VIF and tolerance values, and adjusted odds ratios with 95% CIs from this combined model were reported. All analyses were performed using IBM SPSS Statistics version 21.0 (IBM Corp., Armonk, NY, USA).

## Results

### Characteristics of the study cohort

A total of 25,863 HRT-FET cycles were included in the analysis. The mean serum E2 level before progesterone administration was 343.28 ± 358.26 pg/mL, and the mean P level was 0.18 ± 0.11 ng/mL, resulting in a mean E2/P ratio of 2.52 ± 3.03 (calculated after converting P from ng/mL to pg/mL to match the units of E2). The overall CPR was 54.7% (14,136/25,863), and the LBR was 44.2% (11,441/25,863). Cycles were divided into four groups according to serum E2/P quartiles. Female age, the proportion of secondary infertility, the proportion of patients with polycystic ovary syndrome (PCOS) or multiple infertility factors, and serum E2 levels before progesterone administration increased progressively across the quartiles. In contrast, the proportion of primary infertility, male factor infertility, and serum P levels before progesterone administration decreased progressively (all *P* < 0.01). Body mass index (BMI), indications for infertility (tubal factor, endometriosis), embryo transfer stage, number of embryos transferred, endometrial thickness (both at the start of progesterone and at embryo transfer), and basal E2 levels also differed significantly among groups, although these differences did not follow a consistent increasing or decreasing trend. CPR and LBR showed significant differences across quartiles (both *P* < 0.001), decreasing progressively with increasing E2/P ratios. No significant differences were observed in early spontaneous abortion rates among the quartiles (**Table 1**).

**Table 1:** Patients’ baseline characteristics.

Characteristics	Overall	E2/P Q1	E2/P Q2	E2/P Q3	E2/P Q4	*P*
Cycles (n)	25863	6410	6447	6540	6466	
Age (years)	32.68 ± 5.09	31.47 ± 4.68	32.45 ± 4.90	33.55 ± 5.26	33.23 ± 5.25	0.000**
BMI (Kg/m2)	22.03 ± 2.89	21.96 ± 2.92	22.06 ± 2.90	22.18 ± 2.92	21.91 ± 2.83	0.000**
Infertility type						0.000**
Primary infertility	42.2% (10844/25717)	51.4% (3282/6387)	45.2% (2899/6411)	39.8% (2589/6504)	32.3% (2074/6415)	
Secondary infertility	57.8% (14873/25717)	48.6% (3105/6387)	54.8% (3512/6411)	60.2% (3915/6504)	67.7% (4341/6415)	
Indication for infertility						
Tubal	65.5% (16937/25863)	66.0% (4233/6410)	66.5% (4290/6447)	63.5% (4154/6540)	65.9% (4260/6466)	0.001**
EMT	14.4% (3714/25863)	14.5% (929/6410)	14.9% (963/6447)	14.9% (977/6540)	13.1% (845/6466)	0.006**
PCOS	10.6% (2754/25863)	7.9% (507/6410)	9.1% (588/6447)	11.2% (730/6540)	14.4% (929/6466)	0.000**
Male factor	6.7% (1739/25863)	8.7% (558/6410)	7.3% (469/6447)	6.0% (394/6540)	4.9% (318/6466)	0.000**
Multiple factors	17.0% (4401/25863)	15.9% (1017/6410)	16.7% (1077/6447)	17.3% (1134/6540)	18.1% (1173/6466)	0.005*
Stage of embryo transfer						0.007*
Cleavage-stage embryo	76.9% (19894/25863)	76.2% (4885/6410)	75.8% (4889/6447)	77.7% (5079/6540)	78.0% (5041/6466)	
Blastocyst	23.1% (5969/25863)	23.8% (1525/6410)	24.2% (1558/6447)	22.3% (1461/6540)	22.0% (1425/6466)	
Number of embryos transferred						0.000**
One embryo	14.2% (3668/25863)	11.8% (756/6410)	13.9% (898/6447)	15.9% (1038/6540)	15.1% (976/6466)	
Two embryos	84.2% (21776/25863)	87.2% (5587/6410)	84.6% (5452/6447)	82.1% (5367/6540)	83.0% (5370/6466)	
Three embryos	1.6% (419/25863)	1.0% (67/6410)	1.5% (97/6447)	2.1% (135/6540)	1.9% (120/6466)	
Endometrial thickness: starting progesterone(mm)	8.21 ± 1.31	8.32 ± 1.18	8.46 ± 1.28	8.47 ± 1.34	7.59 ± 1.24	0.000**
Endometrial thickness: embryo transfer (mm)	8.82 ± 1.47	9.08 ± 1.31	9.13 ± 1.38	9.05 ± 1.44	8.00 ± 1.42	0.000**
Basal serum E2 (pg/ml)	33.14 ± 15.08	32.06 ± 14.64	32.95 ± 15.10	33.86 ± 15.58	33.68 ± 14.93	0.001**
Serum E2 before progesterone administration (pg/ml)	343.28 ± 358.26	99.60 ± 51.83	134.50 ± 73.51	225.70 ± 176.56	911.96 ± 184.61	0.000**
Serum P before progesterone administration (ng/ml)	0.18 ± 0.11	0.27 ± 0.15	0.16 ± 0.09	0.13 ± 0.08	0.15 ± 0.06	0.000**
E2/P	2.52 ± 3.03	0.40 ± 0.14	0.86 ± 0.15	1.68 ± 0.44	7.11 ± 2.72	0.000**
Clinical pregnancy rate	54.7% (14136/25863)	60.6% (3884/6410)	57.7% (3722/6447)	52.7% (3445/6540)	47.7% (3085/6466)	0.000**
Live birth rate	44.2% (11441/25863)	50.6% (3245/6410)	47.1% (3037/6447)	42.6% (2788/6540)	36.7% (2371/6466)	0.000**
Early spontaneous abortion rate	6.4% (1644/25863)	6.3% (404/6410)	6.6% (423/6447)	6.2% (406/6540)	6.4% (411/6466)	0.867

E2/P, estradiol to progesterone ratio; Q, quartiles; BMI, body mass index; EMT, endometriosis; PCOS, polycystic ovary syndrome; E2, estradiol; P, progesterone; **p < 0.01. There is overlap among the clinical indications for infertility.

### Factors influencing CPR and LBR during HRT-FET cycles

Multivariate logistic regression analysis was performed to evaluate factors associated with CPR and LBR (**Table 2**). Higher E2/P ratios (Q2, Q3, and Q4 vs. Q1), advanced female age, and tubal factor infertility were identified as negative predictors of both CPR and LBR. Positive predictors included blastocyst transfer, a higher number of embryos transferred, increased endometrial thickness, PCOS, and male factor infertility. BMI was a negative predictor for LBR but not for CPR. Basal E2 levels, E2 and P levels prior to progesterone administration, E2/P ratio as a continuous variable, infertility type (primary vs. secondary), and other infertility indications (endometriosis and multiple factors) were not retained in the final multivariate model due to lack of statistical significance.

**Table 2:** Factors associated with CPR and LBR according to logistic regression analysis.

Variables	CPR		LBR	
	Adjusted OR (95% CI)	*P*	Adjusted OR (95% CI)	*P*
E2/P Quartile Grouping (Q2/Q1)	1.105 (1.000–1.222)	0.050	1.146 (1.041–1.261)	0.005**
E2/P Quartile Grouping (Q3/Q1)	1.210 (1.095-1.338)	0.000**	1.186 (1.077-1.307)	0.000**
E2/P Quartile Grouping (Q4/Q1)	1.415 (1.278–1.567)	0.000**	1.500 (1.358–1.656)	0.000**
Age	1.088 (1.080–1.096)	0.000**	1.091 (1.082–1.099)	0.000**
BMI	–	–	1.029 (1.017–1.041)	0.000**
Infertility diagnosis (Secondary infertility/Primary infertility)	–	–	–	–
Indication for infertility- Tubal	1.267 (1.137–1.411)	0.000**	1.226 (1.106–1.359)	0.000**
Indication for infertility- EMT	–	–	–	–
Indication for infertility- PCOS	0.728 (0.653–0.811)	0.000**	0.779 (0.703–0.862)	0.000**
Indication for infertility- Male factor	0.721 (0.608–0.854)	0.000**	0.707 (0.603–0.828)	0.000**
Indication for infertility- Multiple factors	–	–	–	–
Stage of embryo transfer (Blastocyst/Cleavage-stage embryo)	0.504 (0.461–0.551)	0.000**	0.567 (0.522–0.616)	0.000**
Number of embryos transferred	0.599 (0.540–0.665)	0.000**	0.670 (0.604–0.743)	0.000**
Endometrial thickness-starting progesterone	0.895 (0.870–0.920)	0.000**	0.905 (0.881–0.930)	0.000**
Basal E2	–	–	–	–
E2 before progesterone administration	–	–	–	–
P before progesterone administration	–	–	–	–
E2/P	–	–	–	–

CPR, clinical pregnancy rate; LBR, live birth rate; OR, odds ratio; CI, confidence interval; E2/P, estradiol to progesterone ratio; Q, quartiles; BMI, body mass index; EMT, endometriosis; PCOS, polycystic ovary syndrome; E2, estradiol; P, progesterone; “-” indicates that the variable is not included in the model; **p < 0.01. The final regression model excluded endometrial thickness at embryo transfer due to collinearity with endometrial thickness at the start of progesterone administration.

### Relationship between E2/P ratio and CPR and LBR

The association between the E2/P ratio and pregnancy outcomes was further assessed using multivariate logistic regression (**Table 3**). When treated as a categorical variable with Q1 as the reference, higher E2/P ratios (Q2–Q4) were associated with significantly lower CPR and LBR in the unadjusted model. In both the partially adjusted model (adjusting for maternal age, BMI, infertility type, and infertility indications—tubal, PCOS, male factor) and the fully adjusted model (further adjusting for embryo transfer stage, number of embryos transferred, and endometrial thickness at the start of progesterone), higher E2/P ratios (Q3 and Q4) remained negatively associated with CPR and LBR. Q2 showed a negative impact only on LBR in the fully adjusted model. In all models, the *P* for trend values for both CPR and LBR were <0.001, indicating a significant downward trend in pregnancy outcomes with increasing E2/P ratios. To further assess the robustness of these findings, a sensitivity analysis using tertile grouping (**Supplementary Table 2**) was performed. Higher E2/P tertiles were consistently associated with lower CPR and LBR in the fully adjusted model, aligning with the results from the quartile-based analysis and supporting the stability of the association across different grouping approaches.

**Table 3:** Association between E2/P ratio and pregnancy outcomes in different models.

Pregnancy outcomes	Crude modela a		Adjusted model I b		Adjusted model II c			
	OR (95% CI)	P	OR (95% CI)	P	OR (95% CI)	P	IPTW-OR (95% CI)	IPTW-P
CPR								
E2/P Q1	Reference		Reference		Reference		Reference	
E2/P Q2	1.126(1.049-1.208)	0.000**	1.041(0.959-1.130)	0.343	1.063(0.979-1.155)	0.146	1.058(1.000-1.120)	0.05
E2/P Q3	1.381(1.288-1.481)	0.000**	1.160 (1.068-1.259)	0.000**	1.182(1.087-1.285)	0.000**	1.158 (1.095-1.225)	0.000**
E2/P Q4	1.685(1.571-1.807)	0.000**	1.577(1.453-1.712)	0.000**	1.448(1.331-1.576)	0.000**	1.333 (1.261-1.409)	0.000**
*P* for trend	0.000**		0.000**		0.000**			
LBR								
E2/P Q1	Reference		Reference	–	Reference	–		
E2/P Q2	1.151(1.074-1.234)	0.000**	1.075(0.994-1.163)	0.070	1.098(1.014-1.188)	0.021*	1.093(1.035-1.153)	0.001**
E2/P Q3	1.380(1.287-1.479)	0.000**	1.144(1.057-1.239)	0.000**	1.166(1.076-1.264)	0.000**	1.141(1.081-1.205)	0.000**
E2/P Q4	1.771(1.650-1.900)	0.000**	1.624(1.499-1.759)	0.000**	1.507(1.388-1.636)	0.000**	1.388 (1.315-1.464)	0.000**
*P* for trend	0.000**		0.000**		0.000**			

a, no adjustments for other covariates; b, adjusted for female age, BMI, infertility type, indication for infertility-tubal, PCOS, and male factor; c, adjusted for all covariables in model I plus Stage of embryo transfer, Number of embryos transferred, and Endometrial thickness: starting progesterone(mm). Inverse probability of treatment weighting, IPTW. A corresponding IPTW analysis was additionally performed based on the covariates included in model c, and IPTW-weighted estimates are presented. *p < 0.05, **p < 0.01.

To further evaluate the robustness of these findings, inverse probability of treatment weighting (IPTW) was applied. The IPTW-adjusted analyses produced effect estimates that were consistent with those from the conventional multivariable models. Compared with Q1, the IPTW-adjusted odds ratios for CPR were 1.058 (95% CI: 1.000–1.120) for Q2, 1.158 (95% CI: 1.095–1.225) for Q3, and 1.333 (95% CI: 1.261–1.409) for Q4, with statistically significant P values for Q3 and Q4. For LBR, the IPTW-adjusted odds ratios were 1.093 (95% CI: 1.035–1.153) for Q2, 1.141 (95% CI: 1.081–1.205) for Q3, and 1.388 (95% CI: 1.315–1.464) for Q4 (all P < 0.01). These IPTW results closely aligned with the fully adjusted models and supported the observed trend of decreasing CPR and LBR across higher E2/P quartiles (**Table 3**).

In addition, to assess whether thin endometrium in the highest E2/P subgroup might influence the observed associations, a sensitivity analysis restricting endometrial thickness to 7–14 mm was performed. As shown in **Supplementary Table 1**, the associations between increasing E2/P quartiles and lower CPR and LBR remained directionally consistent and statistically significant, with effect estimates similar to those in the primary analyses. These findings suggest that the observed associations were not explained by the presence of thin endometrium and support the robustness of the results across clinically relevant endometrial thickness ranges.

Finally, to directly compare the prognostic contribution of the E2/P ratio with that of serum E2 within the same cohort, we constructed an augmented multivariable model in which both E2 quartiles and E2/P quartiles were simultaneously included together with all covariates from the fully adjusted model (**Supplementary Table 3**). In this combined model, serum E2 was no longer significantly associated with either CPR or LBR (all P > 0.05), whereas the E2/P ratio remained significantly associated with both outcomes across quartiles (all P < 0.01 for Q3–Q4). Collinearity diagnostics demonstrated acceptable levels of correlation between E2 and the E2/P ratio (VIF = 2.821; tolerance = 0.354), indicating that multicollinearity did not materially affect model stability. These combined-model results further support the robustness of the association between higher E2/P ratios and lower CPR and LBR in HRT-FET cycles.

## Discussion

In this large retrospective cohort of 25,863 HRT-FET cycles, higher pre-progesterone E2/P ratios were consistently associated with lower clinical pregnancy and live birth rates. These associations remained stable across multiple analytic approaches, including fully adjusted multivariable models, IPTW analyses, and two complementary sensitivity analyses—one using tertile grouping and another restricting endometrial thickness to 7–14 mm. All analytic strategies demonstrated a similar graded decline in pregnancy outcomes with increasing E2/P ratios, supporting the robustness of the observed associations across different modeling and grouping methods.

The E2/P ratio reflects the physiological transition between the proliferative and secretory phases of endometrial development. Under normal conditions, estradiol stimulates proliferation of epithelial and stromal cells and induces progesterone receptor expression, thereby priming the endometrium for subsequent progesterone-driven secretory transformation (22–24). Progesterone then initiates glandular secretion and stromal decidualization, processes essential for embryo implantation and early pregnancy maintenance (5). A substantial body of molecular evidence has shown that this transition is accompanied by coordinated changes in key endometrial receptivity markers—such as integrin β3 and leukemia inhibitory factor (LIF)—as well as broader transcriptomic shifts that define the window of implantation (25–27). When estradiol levels are disproportionately elevated relative to progesterone, excessive estrogen exposure may downregulate or desensitize progesterone receptors, disrupt endometrial differentiation, and impair the establishment of the implantation window (2, 6, 28, 29). Furthermore, dysregulation of steroid hormone balance has been associated with altered expression of adhesion molecules, cytokines, and progesterone-responsive genes that are critical for epithelial–stromal crosstalk during implantation (30, 31). This mechanistic framework supports our observation that an elevated E2/P ratio—indicating a relative predominance of estrogenic signaling—correlates with diminished implantation and live birth potential in HRT-FET cycles.

Unlike prior studies that focused solely on absolute hormone concentrations, the present study highlights the clinical relevance of the E2/P ratio as a composite marker of endometrial hormonal equilibrium. Optimal endometrial receptivity depends not only on adequate hormone levels but also on their proportional relationship and temporal coordination (23). The progressively decreasing CPR and LBR across increasing E2/P quartiles likely mirror the physiological consequences of disrupted estrogen–progesterone interplay. Interestingly, early miscarriage rates did not differ significantly among quartiles, suggesting that the detrimental effects of an elevated E2/P ratio primarily affect implantation rather than post-implantation maintenance of pregnancy.

Our findings align with and extend previous research on hormonal dynamics during endometrial preparation. We previously reported that elevated serum estradiol concentrations before progesterone initiation were independently associated with reduced CPR and LBR (13). Similarly, Zhou et al. reported comparable results (11), collectively supporting the concept that supraphysiological estrogen exposure compromises endometrial receptivity. Building on this prior evidence, the present study further evaluates whether the proportional relationship between estradiol and progesterone provides additional insight beyond the predictive value of estradiol alone. The graded decline in pregnancy outcomes across E2/P quartiles observed in the current study suggests that the E2/P ratio may capture aspects of endocrine disequilibrium not fully reflected by absolute E2 levels. Consistent with this interpretation, an augmented regression model incorporating both serum E2 and the E2/P ratio demonstrated that the ratio remained significantly associated with CPR and LBR, whereas serum E2 did not (**Supplementary Table 3**), indicating that the proportional balance between estradiol and progesterone provides additional information beyond estradiol concentration alone. Furthermore, both the IPTW analyses and the sensitivity analysis restricting endometrial thickness to 7–14 mm yielded results that closely aligned with the fully adjusted models, further supporting the stability of the observed associations and suggesting that they are not attributable to imbalances in baseline characteristics or the presence of thin endometrium. Recent work further supports this view: a study by Zhao et al. found that in fresh embryo transfer cycles, E2/P ratios correlated with pregnancy outcomes within a specific range (15), while a small FET study similarly linked higher E2/P ratios with poorer outcomes (14). However, the physiological interpretation of the E2/P ratio differs fundamentally across cycle types. Fresh cycles involve endogenous corpus luteum activity and dynamic hormonal feedback, whereas HRT-FET cycles rely entirely on exogenous steroids and lack luteal endocrine function. Moreover, Zhao et al. assessed the ratio at OPU + 7—near implantation—whereas our measurement before progesterone initiation reflects the priming phase. These endocrine and temporal differences provide a coherent explanation for the divergent associations across studies. To avoid endogenous hormonal interference, cycles with spontaneous follicular development were excluded based on pre-progesterone hormone screening (P < 1 ng/mL), confirming a hormonally regulated HRT-FET cohort.

Conversely, other investigations, including randomized controlled trials and studies comparing natural, modified natural, and programmed FET protocols (17–20, 32), did not observe significant associations between pre-progesterone hormonal parameters and pregnancy outcomes. These discrepancies likely reflect variations in sample size, patient population, hormonal regimen, and timing of measurement. Our study, with over 25,000 cycles and rigorous multivariable adjustment, provides more robust statistical power and reduces potential confounding. Notably, the study by Chadi et al. found that in HRT-FET cycles, patients with low serum progesterone levels before embryo transfer who received combined subcutaneous and vaginal progesterone support exhibited a significantly higher live birth rate compared to those who received vaginal progesterone alone (33). This finding indicates that individualized adjustment of the estrogen–progesterone balance can directly impact reproductive outcomes. Together with our results, these findings suggest that tailoring hormonal support based on the E2/P ratio may help optimize endometrial preparation and implantation success in HRT-FET cycles.

Interestingly, in our fully adjusted model, the second quartile (Q2) of the E2/P ratio was negatively associated with LBR but not with CPR. This finding suggests that a mild increase in the E2/P ratio may not impair implantation itself but could affect endometrial maintenance or early placentation, thereby influencing live birth potential. One possible explanation is that subtle hormonal disequilibrium may have limited impact on the establishment of implantation but could influence processes occurring after implantation, such as decidual stability, early trophoblast invasion, and the development of early placental structures, which are known to be sensitive to progesterone-driven signaling pathways (34, 35). Furthermore, baseline estradiol levels, pre-progesterone estradiol and progesterone levels, and E2/P ratio as a continuous variable were not retained in the final multivariate models for CPR or LBR, possibly indicating that endometrial receptivity depends more on the interaction and dynamic regulation of these hormones rather than their absolute concentrations. Future mechanistic research should investigate how estrogen–progesterone disequilibrium affects molecular determinants of receptivity, including progesterone receptor signaling, endometrial transcriptomic profiles, and the timing of the implantation window. Although the present findings highlight the potential value of the E2/P ratio as an indicator of hormonal synchrony during endometrial preparation, the optimal clinical approach to modifying this ratio remains uncertain. In current clinical practice, several strategies—such as reducing estradiol exposure when the ratio becomes excessively elevated, adjusting the timing of progesterone initiation, or tailoring progesterone supplementation—have been considered, but their relative efficacy has not been rigorously evaluated. Given the retrospective design of our study, no conclusions can be drawn regarding whether any of these interventions would improve outcomes. Prospective, protocol-based interventional studies—such as hormone-adjustment strategies guided by real-time E2/P monitoring—will be essential to determine whether targeted modulation of this ratio can meaningfully enhance endometrial preparation and subsequent reproductive outcomes.

### Limitations

This study has s everal limitations. First, the retrospective observational design and potential indication-related confounding prevent causal inference. Second, serum hormone levels may not perfectly reflect intra-endometrial hormone activity or receptor expression. Third, hormone measurements were performed prior to progesterone initiation rather than at embryo transfer, limiting our ability to assess dynamic hormonal changes. Fourth, data on total estrogen dose, duration of estrogen exposure, and detailed endometrial pattern or morphology were incomplete and therefore could not be incorporated into the analyses, which may limit a more nuanced characterization of endometrial responsiveness. Additionally, residual confounding from unmeasured factors—such as medication adherence, number of previous transfers, detailed embryo quality scoring, and comorbidities—cannot be excluded despite multivariable adjustment. Finally, our findings are restricted to HRT-FET cycles and may not be generalizable to natural or stimulated cycles. Nevertheless, the large sample size, standardized HRT protocol, and comprehensive confounder adjustment strengthen the validity and clinical relevance of our results.

## Conclusions

This study demonstrates an association between higher pre-progesterone E2/P ratios and lower clinical pregnancy and live birth rates in HRT-FET cycles. These findings suggest that the relative balance between estradiol and progesterone may play a role in endometrial preparation; however, causality cannot be inferred due to the retrospective observational design and the possibility of treatment-related confounding. While the E2/P ratio may serve as a useful marker to better understand endometrial hormonal conditions, whether modifying this ratio can improve clinical outcomes remains uncertain. Prospective, well-controlled studies are needed to determine the clinical utility of incorporating the E2/P ratio into endometrial preparation strategies.

## Data Availability

The raw data supporting the conclusions of this article will be made available by the authors, without undue reservation.
