# Supplementary material for: Impact of estradiol-to-progesterone ratio before progesterone initiation on pregnancy outcomes in frozen embryo transfer cycles with hormone replacement therapy: an analysis of over 25,000 cycles
Source: Front Endocrinol (Lausanne). 2026 Mar 9;17:1755013. doi: 10.3389/fendo.2026.1755013 (PMC13006208; doi:10.3389/fendo.2026.1755013)
Supplement: Supplementary file 1 [file Table1.docx]

**Supplemental table 1. Sensitivity analysis of the association between the E2/P ratio and pregnancy outcomes in fully adjusted models after restricting endometrial thickness to 7–14 mm**

|  | **CPR** | | **LBR** | |
| --- | --- | --- | --- | --- |
|  | **Adjusted OR (95% CI)** | ***p*** | **Adjusted OR (95% CI)** | ***p*** |
| E2/P Q1 | Reference |  | Reference |  |
| E2/P Q2 | 1.063 (0.978–1.155) | 0.151 | 1.096 (1.013–1.187) | 0.023* |
| E2/P Q3 | 1.180 (1.086-1.283) | 0.000** | 1.164 (1.074-1.261) | 0.000** |
| E2/P Q4 | 1.447 (1.329–1.575) | 0.000** | 1.501 (1.382–1.631) | 0.000** |

Note：* p < 0.05, ** p < 0.01.

**Supplemental table 2.** **Sensitivity analysis of the association between tertile-based E2/P ratio groups and pregnancy outcomes in fully adjusted models**

|  | **CPR** | | **LBR** | |
| --- | --- | --- | --- | --- |
|  | **Adjusted OR (95% CI)** | ***p*** | **Adjusted OR (95% CI)** | ***p*** |
| E2/P T1 | Reference |  | Reference |  |
| E2/P T2 | 1.114 (1.036–1.197) | 0.003** | 1.124 (1.049–1.205) | 0.000** |
| E2/P T3 | 1.414 (1.314–1.521) | 0.000** | 1.444 (1.345–1.551) | 0.000** |

Note：T1 (lowest), T2 (middle), T3 (highest). * p < 0.05, ** p < 0.01.

**Supplemental table 3.** **Multivariable associations of E2, the E2/P ratio, and their combined model with pregnancy outcomes**

|  | **CPR** | | **LBR** | |
| --- | --- | --- | --- | --- |
|  | **Adjusted OR (95% CI)** | ***p*** | **Adjusted OR (95% CI)** | ***p*** |
| ^a^E2/P Q1 | Reference | - | Reference | - |
| ^a^E2/P Q2 | 1.063(0.979-1.155) | 0.146 | 1.098(1.014-1.188) | 0.021* |
| ^a^E2/P Q3 | 1.182(1.087-1.285) | 0.000** | 1.166(1.076-1.264) | 0.000** |
| ^a^E2/P Q4 | 1.448(1.331-1.576) | 0.000** | 1.507(1.388-1.636) | 0.000** |
| ^a^E2 Q1 | Reference | - | Reference | - |
| ^a^E2 Q2 | 1.027(0.946-1.116) | 0.522 | 0.986(0.911-1.067) | 0.729 |
| ^a^E2 Q3 | 1.170(1.077-1.272) | 0.000** | 1.131(1.044-1.226) | 0.003** |
| ^a^E2 Q4 | 1.392(1.280-1.514) | 0.000** | 1.423(1.312-1.545) | 0.000** |
| ^b^E2/P Q1 | Reference | - | Reference | - |
| ^b^E2/P Q2 | 1.050(0.965-1.144) | 0.256 | 1.087(1.003-1.179) | 0.043* |
| ^b^E2/P Q3 | 1.150(1.040-1.271) | 0.007** | 1.142(1.037-1.258) | 0.007** |
| ^b^E2/P Q4 | 1.394(1.162-1.673) | 0.000** | 1.326(1.108-1.587) | 0.002** |
| ^c^E2 Q1 | Reference | - | Reference | - |
| ^c^E2 Q2 | 0.976(0.891-1.068) | 0.592 | 0.943(0.865-1.029) | 0.186 |
| ^c^E2 Q3 | 1.068(0.966-1.180) | 0.198 | 1.045(0.949-1.151) | 0.370 |
| ^c^E2 Q4 | 1.036(0.862-1.245) | 0.706 | 1.125(0.939-1.348) | 0.201 |

Note：a, adjusted for female age, BMI, infertility type, indication for infertility-tubal, PCOS, and male factor, stage of embryo transfer, number of embryos transferred, and endometrial thickness: starting progesterone(mm). b, adjusted for all covariables in model a plus E2; c, adjusted for all covariables in model a plus E2/P.
Given the mathematical relationship between serum E2 and the E2/P ratio, collinearity diagnostics were performed. The variance inflation factor (VIF) for E2 and the E2/P ratio was 2.821, with a tolerance of 0.354, indicating no substantial multicollinearity.
